# Beyond opinion classification: Extracting facts, opinions and experiences from health forums

**DOI:** 10.1371/journal.pone.0209961

**Published:** 2019-01-09

**Authors:** Jorge Carrillo-de-Albornoz, Ahmet Aker, Emina Kurtic, Laura Plaza

**Affiliations:** 1 UNED IR & NLP Group, Calle Juan del Rosal, Madrid, Spain; 2 University of Duisburg-Essen, Duisburg, Germany; 3 Department of Human Communication Sciences, University of Sheffield, Sheffield, United Kingdom; University of North Carolina at Charlotte, UNITED STATES

## Abstract

**Introduction:**

Surveys indicate that patients, particularly those suffering from chronic conditions, strongly benefit from the information found in social networks and online forums. One challenge in accessing online health information is to differentiate between factual and more subjective information. In this work, we evaluate the feasibility of exploiting lexical, syntactic, semantic, network-based and emotional properties of texts to automatically classify patient-generated contents into three types: “experiences”, “facts” and “opinions”, using machine learning algorithms. In this context, our goal is to develop automatic methods that will make online health information more easily accessible and useful for patients, professionals and researchers.

**Material and methods:**

We work with a set of 3000 posts to online health forums in breast cancer, morbus crohn and different allergies. Each sentence in a post is manually labeled as “experience”, “fact” or “opinion”. Using this data, we train a support vector machine algorithm to perform classification. The results are evaluated in a 10-fold cross validation procedure.

**Results:**

Overall, we find that it is possible to predict the type of information contained in a forum post with a very high accuracy (over 80 percent) using simple text representations such as word embeddings and bags of words. We also analyze more complex features such as those based on the network properties, the polarity of words and the verbal tense of the sentences and show that, when combined with the previous ones, they can boost the results.

## Introduction and background

Surveys show that patients and carers significantly benefit from social interaction with peers and from the sharing of knowledge, experiences and support. This is particularly prevalent among chronic patients, 23% of whom search the Internet intending to find others with same condition [1], but this also holds for general patient population, as well as for carers [2]. Evaluations of peer-led self-management programmes using social media for several chronic diseases indicate positive outcomes and promise to complement the provision in the given health system [3–5]. This also implies that the volume of data in social media grows continuously. The data contains a vast amount of knowledge shared, which is instantly and freely available and as such of immense value not only for individuals, but also for organizations interested in improving their products and services and practitioners looking for expanding their knowledge.

Accessing this knowledge efficiently is a major challenge. One step towards easier access to relevant information in online health data is *opinion* mining. There are state-of-the-art social media analytic tools for extracting users’ opinions [6–10]. However, although opinions help to determine the different user views on the health topics, their usefulness to the patients and patient carers has limitations. Opinions express a person’s judgment, viewpoint or statement that is conclusive. However, opinions are not always true and to assess their veracity is difficult. However, users who look for information online are also interested in *facts* that make them understand the different complex aspects of the illness. A fact is something that can be checked and backed up with evidence. Facts are a valuable source for patients and carers as they allow e.g. to learn about alternative treatments for a condition or to better understand the medical jargon. In addition to facts, *experiences* of patients or carers are another source of information useful to online health forum users. Such experiences can vary from how a person accepted a particular treatment, how she was diagnosed, what steps she has gone through until e.g. she had a surgery, etc. On these aspects, our manual inspections on chat room discussions about illnesses show that facts and experiences are widely shared among the community members.

We are not aware of any related study that tackled the classification of “facts”, “opinions” and “experiences” in health-related social media data nor provided manually annotated data that can be used to learn models to automatically tackle the classification problem. We are aware of studies within the medical domain which perform classification. However, unlike our focus, they are mainly concerned with classifying research articles by predefined topic classes [11–13], such as those given by the MeSH descriptors [14–16]. Other works aim to classify individual sentences into the IMRAD (Introduction, Method, Results and Discussion) categories [17] or to assign descriptors from the Gene Ontology [18]. These works typically employ machine learning algorithms (particularly, SVM and Naive Bayes) on traditional features such as bags-of-words and bi-grams [11], and only the most advanced works use syntactic structures [19], citation meta-data [20], and concept-based representations [13].

Works dealing with the categorization of user-generated contents in medical forums and social networks are, however, still preliminary. These works mainly deal with the problem of classifying user-generated opinions into “positive” and “negative” [7, 8, 10] or even “neutral” [6], and only few of them deal with the more complex problem of classifying sentiments [9]. Other works explore more specific problems: [21], for instance, propose a binary classification task that aims to determine whether a patient has stopped taking a medication or not, while Zhang et al. [22] identify complementary and alternative medicine-related debates from a popular breast cancer forum.

Outside the health domain, distinction between facts and opinions has been widely studied, the problem being usually formulated as classifying sentences into “subjectives” and “objectives” [23–26]. Works from the sentiment and subjectivity analysis areas usually employ affective lexicons to extract the subjectivity of individual words as well as their polarity, and use them together with the grammatical category of words (noun, verb, adjective, adverb and pronoun) as features for classification. But, to the best of our knowledge, no previous work have addressed the distinction between “facts”, “opinions” and “experiences” in patient-generated contents.

The motivation of this research is therefore, different and more ambitious than that of previous related works. We present some novel and valuable contributions toward the main objective of making online health information accessible to all the stakeholders:

- First, we define a new task that is concerned about the extraction of not only opinions from health-related discussions but also facts and experiences. While traditional works from the sentiment analysis forum divide information in objective (i.e., facts) and subjective (i.e., opinions), this is the first work that makes a distinction between facts and experiences.
- Second, we present a detailed study comparing traditional bags of words with word embeddings, and their combination with other lexical, semantic, network-based and sentiment-based features. In addition, we also propose the use of domain-specific concept embeddings from the UMLS Metathesaurus and evaluate its efficiency.
- Third, we present a manually annotated dataset of online discussions concerning three different diseases: allergies, crohn and breast cancer, that classifies sentences into facts, experiences and opinions. We expect this dataset to be used by other researchers and to encourage work toward the detection of experience-based information in health forums.
- Fourth, we show the existence and importance of experience-based information for patients who consult social media in the search of information about their disease. Actually, our study demonstrates that experiences are nearly as frequent as facts and opinions together.

Overall, we find that it is possible to predict opinions, facts and experiences with a very high accuracy (over 80 percent) by using simple representations based on word embeddings and bags of words. The more complex features such as those based on the network properties, the polarity of words and the verbal tense of the sentences, when combined with the previous ones, boost the results. In contrasts, domain specific features such as UMLS concepts and semantic types have not produced competitive results.

The rest of the article is organized as follows. First, we describe the *eDiseases dataset* and the characteristics of patient-generated textual contents, the machine learning methods and the classification features used in our experiments. Second, we present the evaluation setup and the evaluation results. Third, we discuss the obtained results. Finally, we draw the main conclusions of the study and outline future work.

## Material and methods

Our approach is to evaluate the performance of machine learning algorithms on several types of features in the context of patient-generated text classification. In particular, we aim to classify sentences from online health forum in different classes that reflect different degrees of subjectivity (i.e., “facts”, “opinions” and “experiences”).

Next subsection presents the characteristics of the texts that are likely to be found in health forums, as a previous step to presenting the *eDiseases dataset*, which has been manually developed to evaluate our approach. Next, we discuss the different representations that have been tested (which include lexical, syntactic, network-based and sentiment-based and semantic information) both individually and combined. Then we present the different machine learning approaches that have been evaluated.

### User-generated contents in health forums

Patient-generated contents in forums differ from other types of texts, such as scientific papers or news articles, where classification systems have been extensively tested [13, 27]. First, patient-generated texts use a mixture of generic vocabulary with domain-specific vocabulary. When describing diseases, symptoms and treatments, very specific and technical terms are used. However, it is frequent to find informal conversations among patients that refer to their works, families, etc. As a result, the text must be modeled using both domain-specific and general-purpose knowledge resources.

It is also frequent to find that patients have incorrectly spelled the technical names of treatments and diagnosis, which results in a loss of classification accuracy, since very important concepts are missed. Orthographic and grammatical errors are also common. However, errors are less frequent in health forum than in other social media, such as Twitter, Whatsapp or Facebook.

Another characteristic of patient-generated information in social media is the promotion of negative health behaviors and unscientific therapies, as well as the presence of malicious information and harmful advices. Although the treatment of this type of information is out of the scope of this work, it is an important issue that has an impact on the patient decisions about, for instance, stopping taking their medication or even encouraging them to self-medicate [28].

Finally, one of the most distinctive characteristics of the information exchanged in health forums is the use of a highly emotional language. Patients express their opinions and sentiments, relate their own experiences and ask for advice from people in the same situation. To correctly capture the sentiments expressed in the texts, the use of emotional lexicons is highly recommended [29].

### The eDiseases dataset

As in most relevant works in the area, we used text from different forum in MedHelp (http://www.medhelp.org/) [30]. MedHelp is one of the most popular online health communities. The website includes forums for more than 170 communities, each community devoted to a very specific disease or condition; for example, diabetes—type 1, teen depression, skin cancer, or asthma, just to name a few.

A forum consists of a number of threads or conversations; each conversation is a sequence of comments posted by users (patients). Therefore, the text in the posts is not usually clinical or expert data, but consists in a mix of personal experiences, patients’ concerns and advices, and other more or less contrasted information about their conditions.

To build the dataset, we automatically extracted 10 conversations from three communities: allergies, crohn and breast cancer. We selected a set of diseases that, according to medical expert, show high heterogeneity concerning both the degree of medical understanding of the diseases and the profile of the users:

- **Allergic diseases** include a number of hypersensitivity conditions whose causes are not clearly determined, the symptoms are very different and unspecific, the reactions may vary from very mild to life-threatening, diagnosis is difficult and the treatments and prevention mechanisms still generate some medical controversy. Patients are both men and women of any age.
- **Crohn’s disease** is a chronic disease that may limitate the daily life of the patients and make people feel stressed and depressed. Although symptoms are well defined, it may be very similar to other conditions such as ulcerative colitis. Since there is no cure for this disease and treatments are not always effective, alternative therapies are very common. Crohn’s is more prevalent among adolescents and young adults between the ages of 15 and 35.
- **Breast cancer** is a more well understood disease, where diagnosis and treatments are highly standardized, and the symptoms are usually the same (the presence of a lump that feels different from the rest of the breast tissue) although other more complex symptoms may be present. Patients are mostly women and usually over the age of 40.

The conversations were selected randomly, but we automatically filtered out conversations with less than 10 posts. In total, we extracted 146 posts for allergies, 191 posts for crohn, and 142 posts for breast cancer; which include 983 sentences for allergies, 1780 sentences for crohn, and 1029 sentences for breast cancer, covering a 6 years time interval. We used the GATE tool (GATE: https://gate.ac.uk/) to tokenize the text, label the tokens with their part-of-speech (POS) tags and split it into sentences.

Clear instructions were dictated by a domain expert to three annotators that were guided through a training process. Doubts were consulted and discussed all through the annotation process. Examples were given to clarify the distinction among the different categories:

- A **fact** is something that can be checked and backed up with evidence. A fact can be verified. Examples of factual sentences from the dataset are “*Most of our gloves and supplies are latex free, now*” and “*An upper endoscopy (to look at the esophagus, stomach and small intestine) is another test to rule out Crohn’s as biopsies can be taken of the tissue during the procedure*”. However, it is important to point out that, since users in the networks are not medical experts, some of the “facts” stated by them may not be completely true. When labeling the dataset, we do not accomplish any verification process.
- An **opinion** is a judgment, viewpoint, or statement that is not conclusive. An opinion is not always true and cannot be always proven. Examples of opinionated sentences are “*I think you should see an allergist for some skin and RAST tests to help identify your allergy and any other unknown possibilities, too*” and “*It is not an IBD auto-immune disease and in my opinion, a lazy diagnosis by doctors who cannot be bothered to do proper evaluations*”.
- An **experience** is something someone has lived through and that leaves an impression on her. It is expected to be true (and in this sense is near to the concept of fact), but may be affected by personal impressions and sentiments (so it may include subjective appraisals as in the case of opinions). Examples of sentences describing experiences are “*I was diagnosed with it after my blood work, colonoscopy and biopsies came back positive and after living a nightmare*” and “*I had to take prescription strength Benadryl this morning because of some delayed reaction to something that was making it impossible to sleep because of the reflux*”. We have added this new category to the traditional categorization of facts *vs*. opinions because our manual inspections on chat room discussions about illnesses show that experiences are widely shared among the community members (even more than opinions). Instead, when looking for medical facts patients usually visit contrasted websites such as MedlinePlus or even scientific publications.

It is possible that, when describing an experience, the user also expresses an opinion, so that in the same sentence we find both an experience and an opinion. If a sentence include both types of information, the annotator is asked to label the sentence as “experience”.

In case of doubt, we asked the annotators not to label the sentences. To choose one label for each sentence, we adopted the following guidelines:

1. If two or three annotators assigned the same label to the sentence, then such label was finally assigned.
2. If each of the three annotators assigned a different label to a sentence, then a fourth annotator was asked to select the final label.
3. If a sentence was not labeled by at least two annotators, we preserve the sentence for readiness and labeled it as NOT_LABELED.

Distribution of sentences into classes is shown in Table 1. This table gives a clear idea of the nature of the information that is most commonly found in health-related social network conversations.

**Table 1:** Distribution of sentences into information types (“Facts”, “Experiences” and “Opinions”).

	Facts	Experiences	Opinions
**Allergies**	267	348	271
**Crohn**	273	931	389
**Breast cancer**	225	278	310
**Total**	765	1,557	970

Table 2 shows the average inter-annotator agreement per disease for the different labels. We have calculated agreement for each pair of annotators separately, and then computed the average.

**Table 2:** Percent inter-annotator agreement for the three factuality labels and the three diseases.

	Experience	Opinion	Fact
**Allergies**	86%	69%	65%
**Crohn**	88%	65%	72%
**Breast cancer**	77%	70%	79%
**Average**	84%	68%	72%

Comparing agreement per label, we see that the highest degree of agreement is reached for the “Experience” label, which corresponds to the majority label. The worst agreement was achieved for the “Opinion” label. Experiences seem to be the easiest to identify: they clearly describe something that has happened to the patient, while the boundary between facts and opinions is, in this scenario, not always clear: sometimes, for instance, a patient presents her opinion on a treatment as a refuted fact, but it is just a personal impression that can not be proved. For example, the sentence “*The tumor in your left breast is Grade 1 which is also a good thing*” must be interpreted both as opinion and as a fact.

The eDiseases dataset is available for research in https://zenodo.org/record/1479354.

### Feature types

In this section, we describe the different features used to represent the user-generated text for classification using machine learning algorithms.

#### Lexical features

Lexical features considered include bag of words and noun phrases.

- **Bag of words** (BoW): This is the most widely used feature for text classification. In this representation, each word corresponds to a feature with a weight assigned to it. In our experiments, this weight is the TF*IDF value of the term within the dataset. TF*IDF combines the *term frequency* and the *inverse document frequency* (note that we refer with the word “document” to a sentence) to adjust the frequency of a term for how rarely it is used:
  $$\begin{array}{c} w_{i,j}=tf_{i,j}\times log\left(\frac{N}{df_{i}}\right) \end{array}$$
  where:
  *tf*_*i*,*j*_ is the number of occurrences of *i* in the document *j*;
  *df*_*i*_ is the number of documents containing *i*;
  and *N* is the total number of documents.
- **Noun phrases** (NP): Since previous works have shown that, in some contexts, bag of words representations may discard useful information from the documents [31] due to the fragmentation of the syntactic structures, we have also tested the representation based on noun phrases. To identify noun phrases, we use MetaMap [32]. MetaMap is a tool created by the NLM that maps text to UMLS (Unified Medical Language System: https://uts.nlm.nih.gov/home.html) Metathesaurus concepts [33], and has a facility based on the MedPost tagger [34] that may be used to identify phrases within text. We use as features the TF*IDF values of noun phrases.

#### Semantic features

Semantic representations try to solve some of the limitations of lexical representations [35]. They are expected to better model the meaning of the text by capturing semantic relations between words (such as synonymy) and avoiding word ambiguity. We test two different semantic representations: a fine-grained concept-based representation and a more broad representation based on semantic types.

- **UMLS Metathesaurus concepts** (CUI): We map the text onto UMLS Metathesaurus concepts using the MetaMap tool. MetaMap is invoked using the -y flag that uses the default word sense disambiguation algorithm provided in MetaMap. Finally, we represent the text as the TF*IDF values of the concepts retrieved. Concepts are represented by their CUIs (Concept unique identifiers) to avoid ambiguity issues that may arise when the concept names are used.
- **UMLS Semantic types** (ST): We map the text onto UMLS Semantic types from the UMLS Semantic Network (https://www.ncbi.nlm.nih.gov/books/NBK9679/) and represent it using their TF*IDF values. A semantic type is a broad subject category to which the UMLS Metathesaurus concepts are assigned to. Examples of semantic types are “Disease or syndrome”, “Body Location or Region” and “Chemical”.

#### Positional features (Position)

We have calculated two positional features and tested their joint effect:

- **The position of the sentence within the post**: we hypothesize that the position of the sentence within the post may provide useful insights about the type of information it contains. We have observed, for instance, that the first sentence in the post usually presents the experience of the patient, while the following sentences provide more facts on the disease or ask for advices/opinions on her situation to other patients.
- **The position of the post within the thread**: similarly, the position of the post within the thread may be, a priori, relevant. For instance, we have observed that, while the first post within a thread usually asks for advice concerning any aspect of the management of the corresponding disease, the following posts usually provides opinions and advices about it.

#### Network features (Network)

The following two network features have been jointly considered:

- **Number of replies of the post**: if a post is very popular, this may be an indication of the type of information it deals with (for example, according to previous studies, most people looks for experiences rather than facts and opinions [1]).
- **Is a primary question**: this is a binary feature that is 1 if the sentence belongs to the first post in a conversation, and 0 otherwise. The hypothesis is that the first post in a conversation should be the most informative one, since it poses the initial question; and it is most likely to contain experiences than opinions or facts.

#### Sentiment-based features (SA)

Two features traditionally used to separate facts from opinions have been tested: the number of positive/negative words and the number of adjectives [26].

- **Number of positive/negative words**: number of positive and negative words within the sentence are extracted using three affective lexicons: the General Inquirer [36], SentiSense [37] and SentiStrength [38].
- **Number of adjectives**: grammatical categories of words within a sentence are assigned using Gate (https://gate.ac.uk/). The number of adjectives is used as a classification feature. Although nouns, verbs and adverbs may also be opinion-bearing, adjectives are widely considered as the prototypical expressive subjective elements [26].

#### Grammatical features

Grammatical features are also commonly used as classification features for separating facts and opinions. In particular, the part-of-speech of words in the text and the presence of negations have proven to be useful [26, 39]. Moreover, we want to test if the verb tenses within the sentence may help to predict its information type.

- **Verb tense** (verb): Our hypothesis is that past tense verbs are more frequent when expressing experiences, while facts are most frequently expressed using present tense verbs and advices are usually given using imperative forms. We use as features the number of past, present and imperative verbs in the sentence.
- **Part-of-speech** (POS): grammatical categories of words within a sentence are assigned using Gate. We use as features the number of nouns, verbs, adjectives and adverbs.
- **Negation** (Neg): Negation is implemented as a binary feature that indicates the presence of a negation in the sentence. Detection of negations is done using a negation tokens list from [29].

#### Word embeddings (W2V)

Word embeddings using Word2Vec [40] have been extensively used to measure the semantic similarity between words. Our word embeddings comprise the vectors published by [41]. For each sentence, we created an averaged sum of the word vectors—each word in the sentence was used to obtain its word embeddings and we summed over all the word vectors within the sentence. We use an embedding size of 400 dimensions. We ignored punctuations. The entries in the sum vector were used as features.

### Machine learning algorithms

We have used different learning algorithms implemented in Weka (http://www.cs.waikato.ac.nz/ml/weka/) with the various feature sets described in previous sections. In particular, we have tested support vector machines (implemented in Weka as Sequential Minimal Optimization (SMO)), decision trees (J48), Naive Bayes, linear regression (SimpleLogistic) and meta-classifiers (LogitBoost and AdaBoost, with a C4.5 decision tree as the base learner). Since the SMO algorithm provides the most competitive results, we will only show the classification results for this algorithm. We execute SMO with the Weka default parameters, except for the confidence factor (3.0) and the kernel (NormalizedPolykernel). Parameters were tuned using a held-out development dataset.

## Evaluation and results

This section presents the evaluation setup and the results of the experiments.

### Evaluation setup

To evaluate the ML algorithms on the different combinations of features, we use *accuracy*, *precision*, *recall* and *F-measure*, as traditionally done in supervised classification.

**Accuracy** is the proportion of true results (both true positives and true negatives) among the total number of cases examined, and is computed as follows:

$$\begin{array}{c} Accuracy=\frac{true\;positive+true\;negative}{true\;positive+true\;negative+false\;positive+false\;negative} \end{array}$$ (1)

**F-measure** is the harmonic mean of precision and recall, and is computed as follows:

$$\begin{array}{c} F-measure=\frac{2\times recall\times precision}{recall+precision} \end{array}$$ (2)

where **precision** is defined as:

$$\begin{array}{c} precision=\frac{true\;positive}{true\;positive+false\;positive} \end{array}$$ (3)

and **recall** is:

$$\begin{array}{c} recall=\frac{true\;positive}{true\;positive+false\;negative} \end{array}$$ (4)

Evaluation is performed using 10 cross-fold validation with Weka on the eDiseases dataset. We also show the result of predicting the majority class, in order to detect a common problem for learning algorithms that optimize learning for accuracy (they may be simply predicting the majority class).

### Evaluation baseline

We compare our results with a baseline system that consists in a SVM on a bag-of-words representation. The bag-of-words is calculated as detailed in the Features section. This representation has been found to be a competitive baseline in other similar tasks, such as sentiment analysis [27] and MeSH categorization [13].

### Evaluation results

Tables 3–5 show the average classification performance for the three different diseases. For each disease, five broad groups of experiments are shown, which correspond with the combination of the bag of words (BoW), Noun phrases (NP), UMLS concepts (CUI), UMLS Semantic types (ST) and word embeddings (W2V) features (that we will call *first level or primary features*), with the positional (Position), network-based (Network), sentiment-based (SA) and grammatical features (verb, POS and Neg) features (that we will call *second level or secondary features*).

**Table 3:** Feature comparison for the allergies domain. Results are reported in Accuracy, F-measure, Precision and Recall. Best results are indicated in bold.

Feature	Acc	F-1	Pr	Re
BoW—*Baseline*	56	54,9	55,5	56
Bow+Position	55,9	55	55,3	55,9
Bow+Position+Net	55,7	54,8	54,9	55,6
Bow+Position+Net+SA	55,8	55,2	55,2	55,8
Bow+Position+Net+SA+Neg	55,6	55,2	55,2	55,6
Bow+Position+Net+SA+Neg+verb	56	55,5	55,5	56
Bow+Position+Net+SA+Neg+verb+POS	**56,7**	**56,3**	**56,2**	**56,7**
NP	41,3	40,9	45,4	41,3
NP+Position	40,5	40,8	42,2	40,5
NP+Position+Net	43,6	44	45,6	43,6
NP+Position+Net+SA	47,9	48	48,3	47,9
NP+Position+Net+SA+Neg	51	51,1	51,4	51
NP+Position+Net+SA+Neg+verb	52,1	52,2	52,3	52,1
NP+Position+Net+SA+Neg+verb+POS	**53,9**	**54**	**54,1**	**54**
ST	48,4	48,5	48,8	48,4
ST+Position	48,8	48,7	48,9	48,8
ST+Position+Net	48,3	48,3	48,5	48,3
ST+Position+Net+SA	50,2	20,3	50,5	50,2
ST+Position+Net+SA+Neg	51,1	51,2	51,3	51,1
ST+Position+Net+SA+Neg+verb	51,9	51,9	52	51,9
ST+Position+Net+SA+Neg+verb+POS	**52,4**	**52,4**	**52,6**	**52,4**
CUI	47,8	47,2	47	47,7
CUI+Position	48	47,6	47,2	48
CUI+Position+Net	47,6	47,1	46,9	47,6
CUI+Position+Net+SA	51,9	51,8	51,8	51,9
CUI+Position+Net+SA+Neg	52,6	52,5	52,5	52,6
CUI+Position+Net+SA+Neg+verb	55,9	55,9	55,9	55,9
CUI+Position+Net+SA+Neg+verb+POS	**56,5**	**56,5**	**56,5**	**56,5**
W2V	60,2	59,9	59,9	60,2
W2V+Position	60,5	60,2	60,2	60,5
W2V+Position+Net	60,5	60,2	60,2	60,5
W2V+Position+Net+SA	62,2	61,9	61,9	62,2
W2V+Position+Net+SA+Neg	62,8	62,9	62,9	62,8
W2V+Position+Net+SA+Neg+verb	64,5	64,6	64,6	64,5
W2V+Position+Net+SA+Neg+verb+POS	**65,2**	**65,1**	**65,1**	**65,2**

**Table 4:** Feature comparison for the crohn domain. Results are reported in Accuracy, F-measure, Precision and Recall. Best results are indicated in bold.

Feature	Acc	F-1	Pr	Re
BoW—*Baseline*	67,7	66,9	66,5	67,7
Bow+Position	69,7	68,9	68,5	69,7
Bow+Position+Net	69,7	68,9	68,5	69,7
Bow+Position+Net+SA	70,1	69,3	69	70,1
Bow+Position+Net+SA+Neg	70,1	69,3	69	70,1
Bow+Position+Net+SA+Neg+verb	**71,2**	**70,6**	**74,4**	**71,2**
Bow+Position+Net+SA+Neg+verb+POS	**71,2**	**70,6**	**74,4**	**71,2**
NP	62,6	57,4	59,6	62,6
NP+Position	64,7	60,9	62,2	64,7
NP+Position+Net	64,9	61,2	62,4	64,9
NP+Position+Net+SA	65,2	62,2	62,1	65,2
NP+Position+Net+SA+Neg	64,4	60,9	61,5	64,4
NP+Position+Net+SA+Neg+verb	66,9	64,6	64,7	66,9
NP+Position+Net+SA+Neg+verb+POS	**67,8**	**65,6**	**65,6**	**67,8**
ST	61,7	52,6	55,8	61,8
ST+Position	65,6	60	64,2	65,7
ST+Position+Net	65,9	60,5	64,1	66
ST+Position+Net+SA	65,9	60,5	64,1	66
ST+Position+Net+SA+Neg	65,6	60,8	62,9	65,6
ST+Position+Net+SA+Neg+verb	**67,8**	**64,8**	**65,2**	**67,8**
ST+Position+Net+SA+Neg+verb+POS	67,7	64,8	65	67,7
CUI	65,2	63,3	63,1	65,2
CUI+Position	67,5	66,1	65,9	67,5
CUI+Position+Net	68,1	66,7	66,5	68,1
CUI+Position+Net+SA	67,7	66,4	66,2	67,7
CUI+Position+Net+SA+Neg	67,6	66,3	66	67,6
CUI+Position+Net+SA+Neg+verb	69,4	68,4	68,2	69,4
CUI+Position+Net+SA+Neg+verb+POS	**69,8**	**68,9**	**68,6**	**69,8**
W2V	75,9	75,2	75	75,9
W2V+Position	76,1	75,4	75,3	76,1
W2V+Position+Net	76,1	75,4	75,3	76,1
W2V+Position+Net+SA	77,2	76,5	76,3	77,2
W2V+Position+Net+SA+Neg	77,2	76,5	76,3	77,2
W2V+Position+Net+SA+Neg+verb	**78,1**	**77,5**	**77,5**	**78,1**
W2V+Position+Net+SA+Neg+verb+POS	77,2	76,5	76,3	77,2

**Table 5:** Feature comparison for the breast cancer domain. Results are reported in Accuracy, F-measure, Precision and Recall. Best results are indicated in bold.

Feature	Acc	F-1	Pr	Re
BoW—*Baseline*	62,4	62,1	62,3	62,4
Bow+Position	63,6	63,4	63,5	63,6
Bow+Position+Net	64,1	63,7	64	64,1
Bow+Position+Net+SA	63,6	63,4	63,5	63,6
Bow+Position+Net+SA+Neg	63,6	63,4	63,5	63,6
Bow+Position+Net+SA+Neg+verb	**64,1**	**63,8**	**63,9**	**64,1**
Bow+Position+Net+SA+Neg+verb+POS	64	63,7	63,8	64
NP	51,8	50,2	51,9	51,8
NP+Position	51,9	50,4	52,2	51,9
NP+Position+Net	51,8	50,2	51,9	51,8
NP+Position+Net+SA	55,7	54	56,7	55,7
NP+Position+Net+SA+Neg	55,1	53,5	55,9	55,1
NP+Position+Net+SA+Neg+verb	59,2	57,4	58,8	59,2
NP+Position+Net+SA+Neg+verb+POS	**60,1**	**58,5**	**59,4**	**60,1**
ST	45,6	44,4	45,4	45,6
ST+Position	43,7	42,6	43,3	43,7
ST+Position+Net	48,3	46,4	49,9	48,3
ST+Position+Net+SA	50,6	49,6	51,3	50,6
ST+Position+Net+SA+Neg	49,3	48,4	49,8	49,3
ST+Position+Net+SA+Neg+verb	57,3	56,9	57,1	57,3
ST+Position+Net+SA+Neg+verb+POS	**58,1**	**57,5**	**57,6**	**58,1**
CUI	52	51,5	51,7	52
CUI+Position	52,9	52,2	52,5	52,9
CUI+Position+Net	53,3	52,7	52,8	53,3
CUI+Position+Net+SA	54,5	53,9	54,1	54,5
CUI+Position+Net+SA+Neg	55	54,4	54,6	55
CUI+Position+Net+SA+Neg+verb	60,3	59,8	60,1	60,3
CUI+Position+Net+SA+Neg+verb+POS	**60,6**	**60,2**	**60,4**	**60,6**
W2V	65,8	65,8	65,7	65,8
W2V+Position	65,9	65,9	65,9	65,9
W2V+Position+Net	65,1	65	65,1	65,1
W2V+Position+Net+SA	66,4	66,4	66,4	66,4
W2V+Position+Net+SA+Neg	66,1	66,1	66,1	66,1
W2V+Position+Net+SA+Neg+verb	**66,9**	**66,7**	**66,8**	**66,9**
W2V+Position+Net+SA+Neg+verb+POS	**66,9**	**66,7**	**66,8**	**66,9**

#### Comparison of main feature sets

Concerning the first level of features (BoW, NP, CUI, ST and W2V), Tables 3–5 show that the best performance is obtained when **word embeddings** are used; followed by the traditional feature of **bags of words** (our baseline).

The W2V representation outperforms the baseline for all the three diseases.

The use of **UMLS concepts** (CUI) presents a performance close to the use of bag-of-words baseline, although slightly lower. This is due to MetaMap errors when mapping the text to UMLS. Besides, the fact that we have represented the concepts by their concept unique identifiers rather than by their concept names may have also led to disambiguation errors by MetaMap that have an effect on the classification.

Once again, the results corroborate what previous works have also found [13]: the use of **noun phrases** (NP) causes a significant decrease in performance compared with the use of the bag-of-words baseline. As stated in [19] the reasons seem to be that phrase-based representations have an uneven distribution of feature values and contain many redundant features.

When using the **UMLS semantic types** as classification features the performance is below any other feature, and this is true for the three diseases. It should be reminded that semantic types are very high-level categories to which the Metathesaurus concepts are assigned to. When using semantic types to represent the texts, we find several problems: first, since we are dealing with non-expert generated contents, it is expected that a big deal of the vocabulary will not be domain-specific. This kind of vocabulary is not likely to be mapped to any semantic type, though losing a lot of potentially important information; second, representing the text as a set of semantic types means representing medical concepts at a very high level of generalization that, not only does not have helped to increment recall, but has dramatically decreased precision (and so F-measure). Similar results were found by [13] when using a different generalization strategy (different levels of hypernyms) for the categorization of MeSH descriptors. They found, too, that the higher the generalization, the worse the categorization results. Moreover, it must be mentioned that other previous works have highlighted the existence of errors or inconsistencies in the assignment of semantic types to UMLS concepts, which may be another source of classification errors [42].

Therefore, only the W2V representation beats the BoW baseline, which is, as already told, a very strong one.

Finally, we have experimentally verified that combining the first level features does not produce any improvement.

#### Combination of features

The next group of experiments combines each of the first level features (BoW, NP, CUI, ST and W2V) with the second level features (Position, Network, SA, verb, POS and Neg). A large number of feature combinations could be considered. We have selected a limited set of combinations based on our intermediate research results.

Results in Tables 3–5 show that, for every disease, performance of individual features is usually worse than that of combined features:

- When the **positional features** (the position of sentences within the post and the position of the post within the conversation) are combined with the primary features (BOW, NP, ST, CUI and W2V, respectively), performance increases in most of the cases, but sometimes performance does not change or even decreases. Besides, the increases in performance are not significant.
- The same occurs when the **network-based features** (number of replies of the post and “is a primary question”) are considered. Performance increases in a very small percentage in some cases, but in most of them it stays invariable or even drops.
- When the **sentiment-based features** are added, performance usually increases, but the increment is not significant. Sentiment-based features include the polarity of words and the presence of adjectives. Since the three categories (facts, experiences and opinions) may include positive and negative information, these features are not adequate to discriminate between the three categories.
- The effect of the **negation feature** is quite homogeneous across domains and combinations of features: it decreases performance for nearly all feature combinations and diseases.
- Concerning the **grammatical features** we can observe that the verb feature (which assigns to each sentence the form (present/ past/ imperative) of the verbs within it) usually has a positive influence in classification. This feature always gets an improvement in performance regardless of the primary feature considered and also regardless of the disease. The use of the POS feature (the part of speech of words within the sentence) does not seem to offer any improvement, and performance usually remains constant.
- On the other hand, when **all the features** are combined, the performance is, in general, higher than any individual feature and that any other combination of features.
- It is worth noting that the effect of adding new features is more marked in those primary features that individually produce poor classification results (i.e., ST and CUI), while is nearly insignificant for the W2V feature (i.e., for the best performance individual primary feature).
- Finally, for the W2V feature we have also tested two further combinations of features, W2V + verb and W2V + verb + SA, and found that the results remain similar than those of the other combinations.

**Fig 1:**
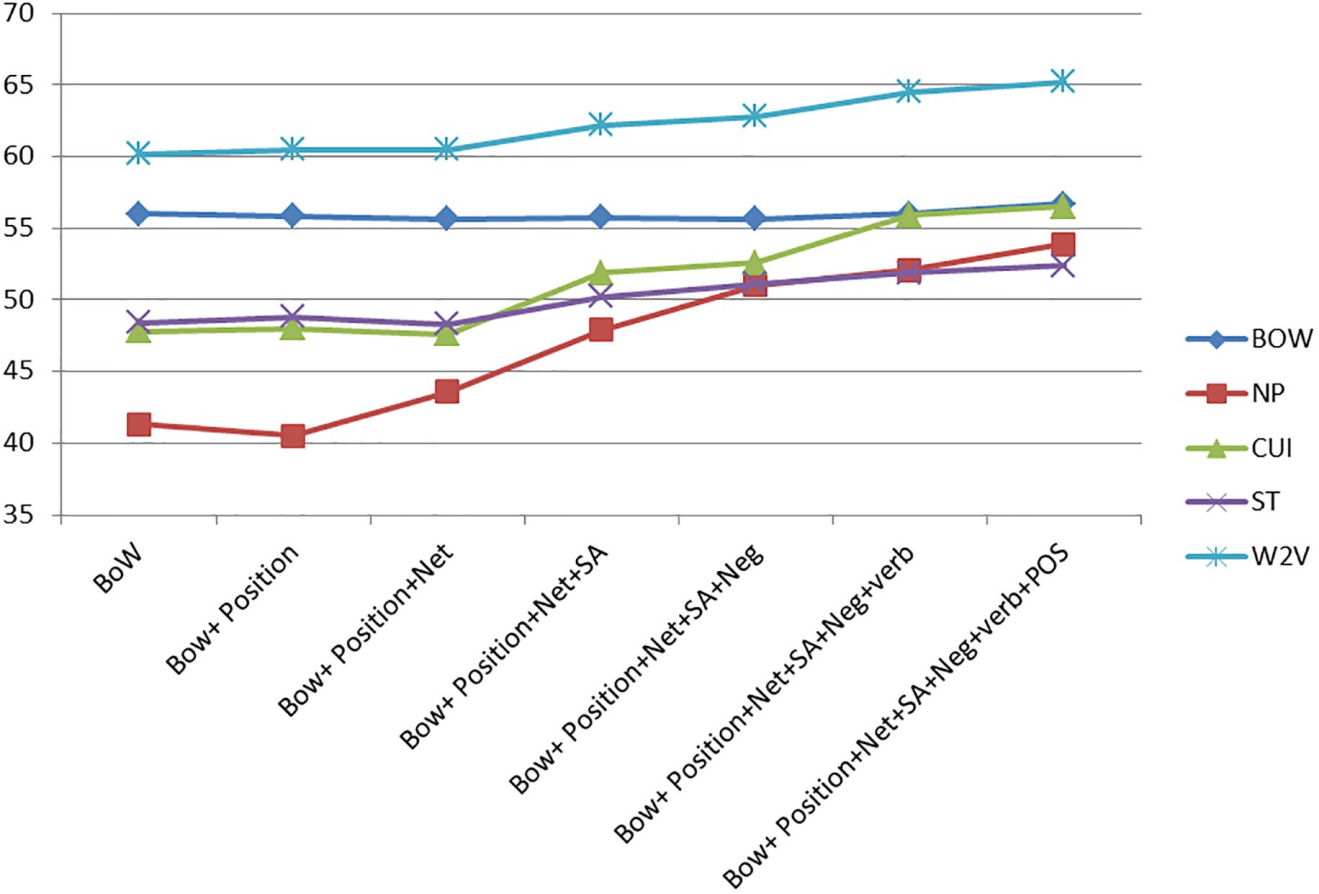
Feature comparison for the allergy domain.

**Fig 2:**
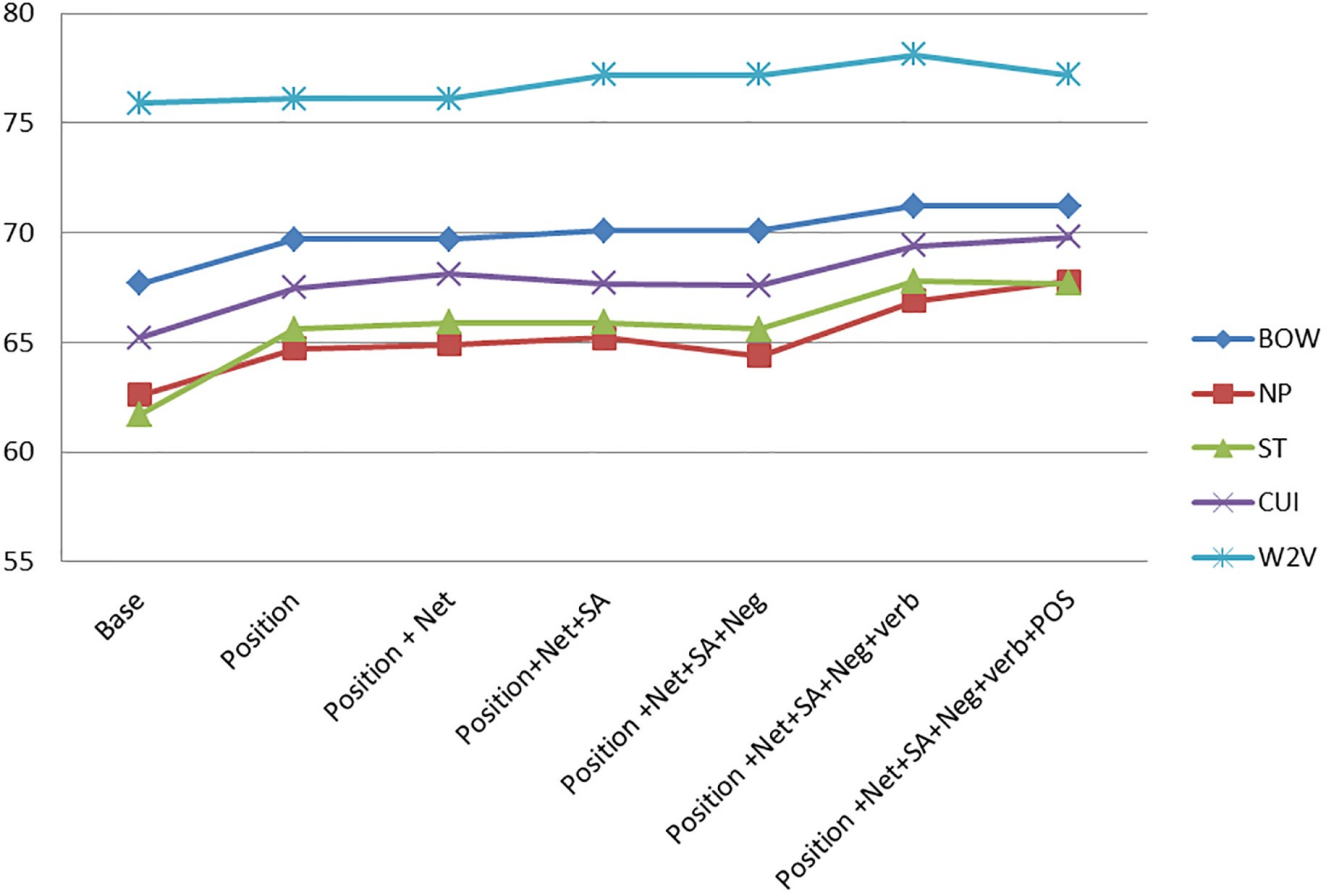
Feature comparison for the crohn domain.

**Fig 3:**
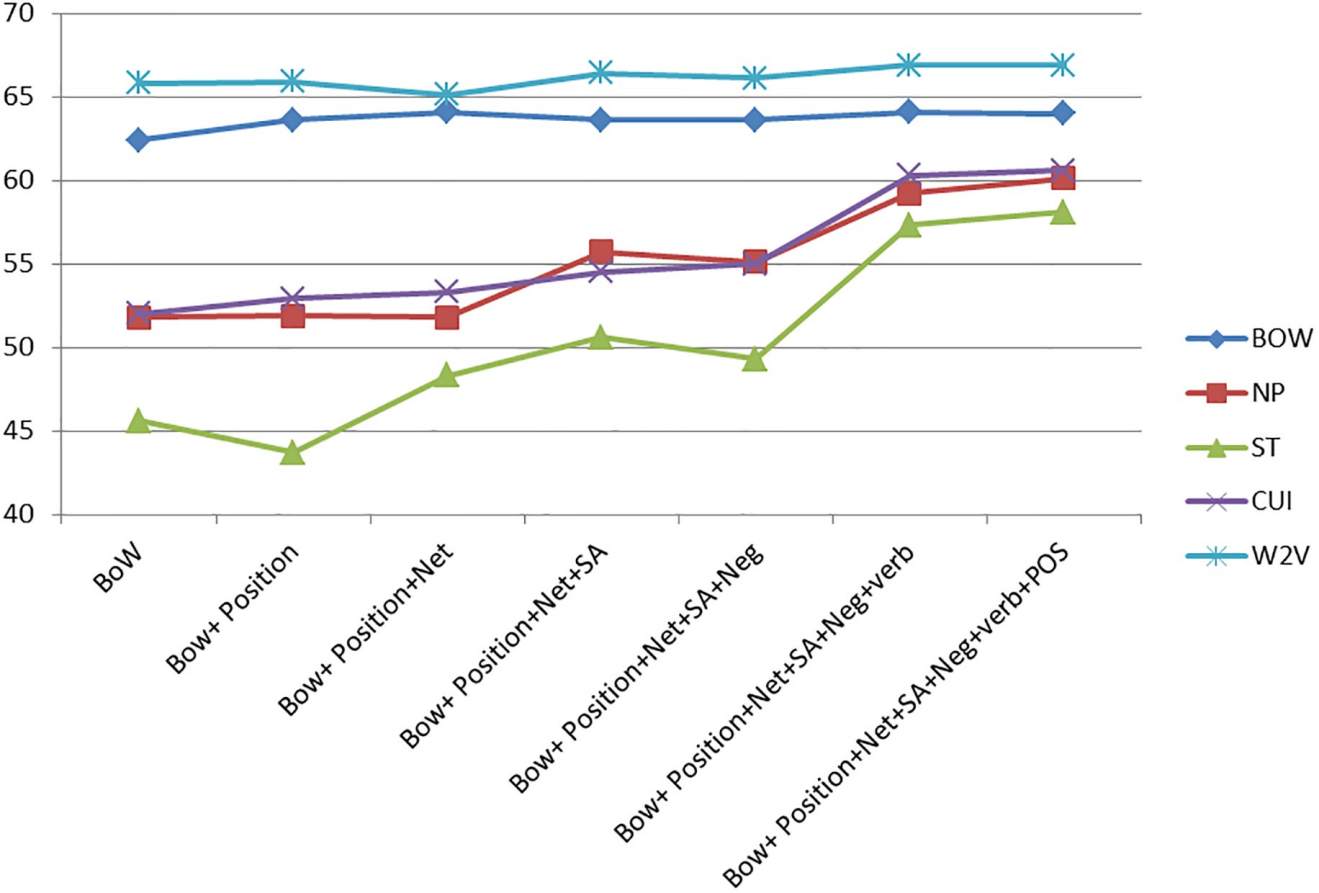
Feature comparison for the breast cancer domain.

The effect of the different features may be better appreciated in Figs 1–3.

#### Comparison among different diseases

In order to facilitate comparison among different diseases, Table 6 shows a summary of the results in previous tables. It may be observed that the best results are obtained for the crohn disease (around 77%) while the results for allergies and breast cancer are similar (around 66%). We also observe that only the W2 and CUI representations (in combination with the secondary features) outperform the baseline. For all the three diseases, the best results are obtained by the word embeddings approach in combination with other features (around 65–78%).

**Table 6:** Comparison between diseases (allergies, crohn, and breast cancer) for the best performance features combinations (F-measure). Best results are indicated in bold.

Feature	Allergies	crohn	Breast cancer
BoW + Position + Network + SA + Neg + verb + POS	56,3	70,6	63,7
NP + Position + Network + SA + Neg + verb + POS	54	65,5	58,5
ST + Position + Network + SA + Neg + verb + POS	52,4	64,8	57,5
CUI + Position + Network + SA + Neg + verb + POS	56,5	68,9	60,2
W2V + Position + Network + SA + Neg + verb + POS	**65,2**	**76,5**	**66,7**
BoW baseline	56	66,9	62,1
Majority baseline	39,3	58,4	38,1

Finally, Table 7 shows the results by class for the W2V classifier and the three diseases. We can observe from the table that, for the three diseases, F-measure is higher for the “Experiences” class than for the remaining two classes (“Facts” and “Opinions”). The reason for these results may be that the “Experiences” class has considerably more instances than the other two in the allergies and crohn diseases, but also that it is easier to learn, since it also gets the best results in breast cancer, where the majority class is “Facts”. In contrast, the worse learned class is “Facts”, which is the class with less instances in the three diseases.

**Table 7:** F-measure by class for the W2V classifier. Best results are indicated in bold.

Class	Allergies	Crohn	Breast cancer
Experiences	**68,5**	**85,3**	**69,8**
Facts	52,9	55,2	60,5
Opinions	55,6	065	066

In the light of these results, it seems that it is possible to improve learning by increasing the number of instances in the training set. However, the results are still well above the majority class, which means that all classes are being reasonably learned.

Nonetheless, to see the effect of the different number of training instances by classes, we have performed a further experiment where the classes has been balanced using the “Re-sample”(subsample) Weka filter. Table 8 shows the results and proves that equilibrating the number of instances per class has a very positive impact in the classification performance, allowing for (a) averaged F-measures over 85% in all three diseases, and (b) similar performance for the three individual classes.

**Table 8:** F-measure by class for the W2V classifier (Resample). Best results are indicated in bold.

Class	Allergies	Crohn	Breast cancer
Experiences	**84,1**	**91,7**	**84,3**
Facts	77,1	78,6	82,0
Opinions	73,8	81,9	83,0
Total	79,1	87,1	83,2

#### Combining the different diseases

So far we have considered each disease in isolation. In the following experiment we study how combining the data from all the three diseases affects the classification. In this way, we aim to understand how adaptable the classifiers are to previously unseen diseases. Table 9 shows the results for the W2V classifier.

**Table 9:** Classification results when data for the three diseases are combined.

Feature	Acc	Pr	Re	F-1
W2V	70,6	70,3	70,7	70,2
W2V—*Resample*	83,4	83,3	83,4	83,4

As it can be observed in Table 9, classification results are slightly better than those obtained for the allergies and breast cancer domains, but slightly worse than those of crohn. These results suggest that it is possible to apply the classifiers to new diseases (where the distribution of data is expected to be different) and still obtain a good performance (over 70%). This means that the features learned are robust to variations across domains.

## Discussion

The experiments show that it is possible to efficiently classify patient-generated contents in facts, opinions and experiences using ML techniques. As mentioned in the introductory section, this distinction is one of the main contributions of the paper and it may be of great interest to help user to quickly find the type of information they are looking for. For instance, they may be looking for experiences and feelings of other patients rather than for contrasted clinical information.

Using simple lexical features, such as bags of words, provides a very strong baseline compared to other more sophisticated features based on semantic representations, such a semantic types (see Tables 3–5). The concept-based representation using UMLS achieves a similar performance than the bag-of-words baseline. More complex grammatical constructions such as noun phrases show lower performance than the bags of words and the conceptual representations. However, the best performance is achieved by the word-to-vector approach. The word embeddings model extends the bags of words model by incorporating context, and provides a significant improvement of classification performance.

In addition, combining the features improves performance especially when the primary or main feature provides poor results (which is the case of the noun phrases and semantic types representations), but the improvement is less important for those primary features that get good performance (see Tables 3–5). Moreover, the combinations of features have a similar performance across the three different diseases.

For nearly all the combinations of features, we also see that the classifier is returning well-balanced precisions and recalls. Besides, although classes are quite unbalanced (especially in the case of the crohn disease), accuracies are quite above the majority class baseline, and F-measures are quite balanced for the different classes (see Table 6).

Regarding differences across diseases, classification performance is considerably higher for the crohn disease than for allergies and breast cancer (≈ 78% vs. ≈ 65%). One of the reasons for this is the different size of the datasets (the crohn dataset having approximately double number of sentences than the other two). However, we have identified other reasons. First, if we look at the distribution of sentences within classes in Table 1, we see that the crohn dataset has a very high percentage of experiences (around 50%) and a low percentage of facts and opinions (around 25% each class). In the light of this, we could think that the “experiences” class is being over-learned, but have shown that performance by class is balanced. Therefore it seems that the “experience” class is easier to classify than the remaining two (see F-measures by class in Table 7). Second, we have noted that in the allergies dataset there are a high number of sentences with non relevant information (i.e., information—facts, opinions and experiences—that is not directly related with allergies) whose vocabulary is very different to that within the relevant sentences, and this may affect classification performance. Moreover, in the allergies dataset information on different types of allergies is mixed, with different symptoms, reactions and treatments, so that again the vocabulary is less homogeneous. Third, in the breast cancer, the proportion of experiences (which seems to be the easier to learn class) is less than in the other diseases.

Supplementary experiments have shown that the performance by class is well balanced for the three disease and that it is possible to significantly increase both average and individual performances by equilibrating the number of instances per classes in the training set.

Finally, our experiments suggest that it is possible to apply the classifiers to previously unseen diseases, without carrying out any adaptation process. This means that the features learned show good cross-domain generalization performance.

## Conclusions

Research in information extraction and classification from medical blogs and discussion forums is gaining increasing attention. Surveys show that patients and carers significantly benefit from social interaction with peers and from the sharing of knowledge, experiences and support [1], but sometimes the huge amount of available information makes it difficult to find that of real interest for users.

Our goal is to make online health information accessible and useful for patients, professionals and researchers. As an important step toward this goal, in this work we have evaluated the feasibility of exploiting lexical, syntactic, semantic, network-based and emotional properties of texts to classify patients-generated contents into “experiences”, “facts” and “opinions”. In this way, we extend the typical sentiment analysis 2-classes task (“opinion” versus “facts”) to include the “experiences” category. Previous studies have noted that patients (particularly those suffering from chronic conditions) use Internet forums especially for searching experiences of others with the same condition [1].

Our results have shown that it is possible to predict this type of information with a very precision, by using simple representations based on word embeddings and bags of words. These combined with more complex features such as those based on the network properties, the polarity of words and the verbal tense of the sentences improve the results. In contrasts, domain specific features such as UMLS concepts and semantic types have not produced competitive results. Moreover, we have manually annotated data for two chronic diseases (allergies and crohn) and breast cancer. We will make this data publicly available for wider research community.

As future work we plan to experiment with the combination of machine learning algorithms, which has previously shown to increase performance [43], and with new categorization tasks (e.g., polarity classification of sentences). We also plan to extend the dataset and to test different types of neural networks for classification.
